# The Interaction of Oxidative Stress Response with Cytokines in the Thyrotoxic Rat: Is There a Link?

**DOI:** 10.1155/2009/391682

**Published:** 2009-03-30

**Authors:** Balahan Makay, Ozer Makay, Cigdem Yenisey, Gokhan Icoz, Gokhan Ozgen, Erbil Unsal, Mahir Akyildiz, Enis Yetkin

**Affiliations:** ^1^Division of Pediatric Rheumatology and Immunology, Department of Pediatrics, School of Medicine, Dokuz Eylul University, 35340 Inciralti, Izmir, Turkey; ^2^Department of General Surgery, School of Medicine, Ege University, 35100 Izmir, Turkey; ^3^Department of Biochemistry, School of Medicine, Adnan Menderes University, 09890 Aydin, Turkey; ^4^Division of Endocrinology, Department of Internal Medicine, School of Medicine, Ege University, 35100 Izmir, Turkey

## Abstract

Oxidative stress is regarded as a pathogenic factor in hyperthyroidism. Our purpose was to determine the relationship between the oxidative stress and the inflammatory cytokines and to investigate how melatonin affects oxidative damage and cytokine response in thyrotoxic rats. Twenty-one rats were divided into three groups. Group A served as negative controls. Group B had untreated thyrotoxicosis, and Group C received melatonin. Serum malondialdehyde (MDA), glutathione (GSH), glutathione reductase (GR), glutathione peroxidase (GPx), and nitric oxide derivates (NO^•^x), and plasma IL-6, IL-10, and TNF-alpha were measured. MDA, GSH, NO^•^x, IL-10, and TNF-alpha levels increased after L-thyroxine induction. An inhibition of triiodothyronine and thyroxine was detected, as a result of melatonin administration. MDA, GSH, and NO^•^x levels were also affected by melatonin. Lowest TNF-alpha levels were observed in Group C. This study demonstrates that oxidative stress is related to cytokine response in the thyrotoxic rat. Melatonin treatment suppresses the hyperthyroidism-induced oxidative damage as well as TNF-alpha response.

## 1. Introduction

Hyperthyroidism is associated with an increased metabolic rate due to increments in the rate of oxygen consumption in target tissues [1]. Acceleration of aerobic metabolism by thyroid hormones enhances the generation of oxidative stress [2]. Oxidative stress is regarded as a pathogenic factor in hyperthyroidism. Clinical and experimental studies reported increased levels of free oxygen radicals and a decreased antioxidant status in thyrotoxicosis [3–6].

Free oxygen radicals are general mediators of signal transduction pathways, which are able to induce cytokine production from various cell types [7–10]. Studies attesting to the role of antioxidants, of which potentially inhibit the activation of oxidant-mediated transcription factors, reported that these antioxidants also prevent the transcriptional activation of inflammatory cytokines [11–13]. These studies suggest that antioxidants may play a role in decreasing the immune response by suppressing the oxidative stress. Since these findings may be of clinical relevance in the thyrotoxic patient, herein we aimed to investigate (1) whether oxidative stress affects the production of cytokines IL-6, IL-10, and TNF-alpha and (2) whether these alterations are inhibited by melatonin-based antioxidative therapy.

## 2. Methods

Twenty-one 12-week-old male Wistar albino rats with a body weight of 250–300 g were included in the study. All were housed in groups of 7 in identical wire-bottomed cages with a 12 hour day-night cycle, at a constant room temperature of 24 ± 1°C. The animals were acclimatized to these conditions for 10 days before the experiment. Standard rat food and water was freely available. All rats were treated according to the UFAW Handbook on the Care and Management of Laboratory Animals (Blackwell, 7th ed, Blackwell Science, Iowa, 1999) and Principles of Laboratory Animal Care (NIH publication no. 86-23, revised 1985). The protocol was approved by Ege University Ethics Committee of Experimental Studies and Ege University , Department of Experimental Surgery.

Rats were randomized to the following groups according to the treatment to which each was subjected and were sacrificed at the end of the 3-weeks treatment:

1. Group A (*n* = 7), the sham group, was not subjected to any procedure, except receiving daily intraperitoneal injections of 0.9% saline solution;
2. Group B (*n* = 7), the untreated (control) group, underwent intraperitoneal L-thyroxine administration for induction of hyperthyroidism, for 21 days and at a daily dose of 0.2 mg/kg. L-thyroxine was dissolved using 0.01 N NaOH, and the final solution was prepared with 0.9% saline solution;
3. Group C (*n* = 7), the treatment group, underwent L-thyroxine as in Group B and besides, received daily (each 24 hours) 3 mg/kg melatonin for 21 days (Ilsan Iltas Laboratories, Istanbul), intraperitoneally.

### 2.1. Laboratory Measurements

All evaluations were done by a single biochemist blinded to the study. Blood was collected through heart puncture to estimate thyroid function tests (TSH, FT_3_, and FT_4_), oxidative stress markers (malondialdehyde [MDA], glutathione [GSH], glutathione reductase [GR], glutathione peroxidase [GPx], and nitric oxide [NO^•^]), and cytokines (IL-6, IL-10 and, TNF-alpha). Blood samples were centrifuged at 2500 g for 5 minutes at room temperature. The serum was removed and stored at −80°C for later studies. Free T_3_ (triiodothyronine) (FT_3_), free T_4_ (thyroxine) (FT_4_), and TSH (thyroid stimulating hormone) analyses were carried out with a chemiluminescent enzyme radioimmunometric assay by using the IMMULATE 2000 automated hormone analyzer (Diagnostic Products, Los Angeles, Calif, USA). The reproducibility of the methods, established before the study by conducting 2 additional independent experiments, was good with clear normative limits and a 95% agreement.

### 2.2. Measurement of MDA

 Lipid peroxidation (LPO) is frequently investigated in biomedical research, and the assays for thiobarbituric acid-reactive substances (TBARSs) are more widely used than any other index of LPO in biological samples. Thiobarbituric acid reacts with LPO aldehydes, such as malondialdehyde (MDA). Therefore, assessment of TBARS is a useful index of oxidative deterioration and LPO determination in body fluids. MDA levels were determined spectrophotometrically at 532 nm by the method of Ohkowa et al. [14]. MDA formed a colored complex in the presence of thiobarbituric acid, which was detectable by measurement of absorbance at 532 nm. Absorbance was measured with Shimadzu UV-160 spectrophotometer. 1,1′,3,3′ tetraethoxypropane was used as a standard. Levels were calculated as nmol/mL.

### 2.3. Measurement of GSH

Total glutathione content was measured according to the method of Tietze [15]. In brief, 0.5 mL sample or standard solution was mixed with 0.25 mL of 1 mol/L sodium phosphate buffer (pH 6.8) and 0.5 mL 5-5′-dithiobis-(2-nitrobenzoic acid) (DTNB, 0.8 g/L in the phosphate buffer) for 5 minutes. Then, the absorbance was measured at 412 nm using a Shimadzu UV-160 spectrophotometer. The GSH concentration was determined using standard aqueous solutions of GSH. Results were expressed as mg/dL.

### 2.4. Measurement of GR

Glutathione reductase was assayed by following the oxidation of NADPH at 340 nm at 37°C [16]. The reaction was initiated by the addition of 50 *μ*L supernatant to 1 mL of assay mixture containing 50 mmol/L Tris, pH 7.6, 100 *μ*mol/L Na_2_EDTA, 4 mmol/L GSSG, and 120 *μ*mol/L NADPH. A blank cuvet was prepared in which the sample was replaced with water. The reaction was linear for 2 to 3 minutes. Hemoglobin in erythrocyte lysates was estimated using the method of Drabkin and Austin [17]. Activity in samples was normalized for hemoglobin content and is expressed in U/g Hb.

### 2.5. Measurement of GPx

Glutathione peroxidase activity was determined by a minor modification of the method of Paglia and Valentine [18]. A 50 *μ*L supernatant was transferred to a 1 mL quartz cuvet, containing 950 *μ*L of the reaction mixture (Tris buffer, 50 mmol/L, pH 7.6, containing per liter, 1 mmol of Na_2_EDTA, 2 mmol of reduced glutathione, 0.2 mmol NADPH, 4 mmol sodium azide, and 1000 U of glutathione reductase). The mixture was incubated 5 minutes in 37°C. Then, the reaction was initiated by adding 25 *μ*L of H_2_O_2_, 8.8 mmol/L (% 30). The decrease in NADPH absorbance at 340 nm was followed for 3 minutes. The nonenzymic reaction rate (blank) was determined by substituting water for the supernatant. The decrease in NADPH absorbance was recorded. Hemoglobin was evaluated as described above. Activity in samples was normalized for hemoglobin content and is expressed in U/g Hb.

### 2.6. Measurement of NO^•^ Derivative Content

 NO^•^x (nitrite + nitrate) was assayed by a modification of cadmium-reduction method as mentioned by Navarro-Gonzàlves [19]. The nitrite produced was determined by diazotization of sulphanilamide and coupling to naphthlethylene diamine. For the measurement of NO^•^x, a 400 *μ*L sample was denatured by adding 80 *μ*L 30% ZnSO_4_ solution, stirring and then centrifuging at 10 000 xg for 20 minutes at 4°C. First, we activated Cd granules using CuSO_4_ solution in glycine-NaOH buffer. Then, 100 *μ*L of deproteinized samples and standards were added. This reaction, using pretreatment of samples to reduce nitrate to nitrite, can be accomplished by catalytic reactions using enzyme or Cd. The samples were analyzed spectrophotometrically using a microplate reader and quantified automatically against KNO_3_ standard curve. Results were expressed as *μ*M/L.

### 2.7. Measurement of IL-6

Plasma IL-6 levels were determined via a commercial rat ELISA kit (IBL Co., Ltd. Hamburg, Germany), catalogue and lot number 17196. The test results were calculated by bioelisa reader ELx800 using standard curve. The kit measurement range was 11.72–750 pg/mL. Interassay values were calculated for three different measurement values (34.83, 71.71, and 293.31 pg/ml), and CV values were found to be 2.3%, 1.9%, and 1.7%, respectively, (*n* = 10). Besides, intra-assay values determined three different measurement values (33.57, 70.45, and 293.45 pg/mL), and CV values were found to be 8.8%, 7.7%, and 4.9%, respectively, (*n* = 10). We did not use haemolysed samples.

### 2.8. Measurement of IL-10

IL-10 levels were determined via a commercial human ELISA kit (IMMUNOTECH; Beckman Coulter Company, Marseille Cedex, France). Test results were calculated by bioelisa reader ELx800 using standard curve. The kit measurement range was 0–2000 pg/ml. Kits' performance characteristics were performed. Intra-assay precision was determined by assaying sera 10 times, and CVs ranged between 3.3 and 4.0%. Interassay precision was determined by assaying samples 10 times in independent assays. CVs ranged between 5.6 and 8.6%. We did not use haemolysed samples.

### 2.9. Measurement of TNF-Alpha

TNF-*α* levels were determined via a commercial rat ELISA kit (IBL Co., Ltd. Hamburg , Germany), catalogue number 17194. Test results were calculated by bioelisa reader ELx800 using standard curve. The kit measurement range was 0.39–25 ng/mL. Kits' performance characteristics were performed. Interassay values were calculated for three different measurement values (0.36, 1.69, and 7.03 ng/mL), and CV values were found to be 8.8%, 3.0%, and 1.0%, respectively, (*n* = 10). Besides, intra-assay values determined three different measurement values (0.33, 1.69, and 7.03 ng/mL), and CV values were found to be 8.8%, 3.0%, and 1.0%, respectively, (*n* = 10). Besides, intra-assay values determined three different measurement values (0.33, 1.68, and 6.7 ng/mL), and CV values were found to be 9.1, 9.5, and 4.3, respectively, (*n* = 10). We did not use haemolysed samples.

### 2.10. Statistical Analysis

All analyses were performed by using SPSS Statistical Analysis software (release 10.0, Chicago, Ill, USA). All data are expressed as mean ± standard deviation. To search difference between the groups, Kruskal-Wallis one-way analysis was used. Depending on the homogeneity of the variances, Mann-Whitney *U*-test and Bonferonni post hoc test were used in pair comparison. Correlations between oxidative stress parameters and serum concentrations of cytokines were studied by regression analysis. *P*-values of less than .05 were considered to be significant.

## 3. Results

### 3.1. Thyroid State

The thyroid state of different groups of animals was characterized by the data reported in Table 1. The values indicate significant rises in FT_3_ and FT_4_ levels after L-thyroxine administration (*P* < .01). It was also found that melatonin treatment significantly decreased the concentrations of FT_3_ and FT_4_ when compared to rats with hyperthyroidism in the untreated group (*P* < .01).

### 3.2. Oxidative Stress and Response to Oxidative Stress

MDA, GSH, GR, GPx, and NO^•^x levels of control and treatment groups are presented in Table 2. Comparison among the groups revealed that MDA, GSH, and NO^•^x levels in hyperthyroid rats were significantly higher than the negative control and treatment groups (*P* < .01).

### 3.3. Plasma Cytokine Concentration

Levels of both IL-10 and TNF-alpha were significantly enhanced after L-thyroxine administration (*P* < .01), while the rise in IL-6 was insignificant (*P* < .05). Melatonin treatment was associated with significant decreased TNF-alpha levels (*P* < .01) (Table 3). There was a significant correlation between IL-10 concentration and both oxidative stress parameters MDA and NO (*r* = 0.48, *P* < .05 and *r* = 0.39, *P* < .01, resp.). A significant correlation also existed between serum TNF-alpha and MDA (*r* = 0.49, *P* < .01) as well as TNF-alpha and NO level (*r* = 0.69, *P* < .01). No correlations were found between TNF-alpha and thyroid hormone levels (*P* > .05).

## 4. Discussion

The findings of the study suggest that (1) thyrotoxicosis stimulates the oxidative stress response and the production of inflammatory cytokines (IL-10 and TNF-alpha), (2) there is a relationship between oxidative stress parameters and inflammatory cytokines, (3) melatonin prevents the increase in FT_3_ and FT_4_ caused by chronic T_4_ injection, and (4) melatonin-based antioxidative treatment inhibits thyroid hormone induced increases in MDA, GSH, NO^•^, and TNF-alpha levels. Meanwhile, to our knowledge, the latter observation, that melatonin acts on TNF-alpha levels, seems novel. We hypothesized that reactive oxygen intermediates, that were induced by thyrotoxicosis, act as a signal for the release of cytokines like IL-6, IL-10, and TNF-alpha. We documented the increased oxidative stress and antioxidant system capacity, together with response to allopurinol, in different time intervals, in another paper, which is soon going to be published elsewhere.

Previous studies have provided evidence that free radicals increase during hyperthyroidism [3–6]. Whatever the pathways are, it is clear that T_3_-induced liver free-radical generation occurs in concomitance with enhanced respiratory burst activity and chemiluminescent response in both experimental and human studies [20, 21]. The evidence that oxidative stress occurs related to thyroid hormone induction is supported in the current study, since we have demonstrated significant increases in oxidative stress parameters like MDA and NO^•^x. Although some modalities have been suggested to prevent the development of oxidative stress during thyrotoxicosis, no management has yielded promising uniform success, clinically [22–24].

Oxidative stress is paralleled by impaired antioxidant mechanisms; glutathione is one of these important systems [25]. In one of the few studies related to GSH in hyperthyroidism, GSH was stimulated in an experimental model [26]. In these studies, it was concluded that free radicals appear to stimulate GSH production in prolonged oxidative stress. This explanation seems plausible, since biochemical data of our study showed an increase in GSH.

Several previous reports have tried to address the contribution of thyrotoxicosis to the rise in inflammatory cytokines such as IL-1, IL-6, IL-10, and TNF-alpha [13, 27–34]. Although controversial results, almost all these studies suggest that cytokines may play important roles in the process of hyperthyroidism. The only report investigating the effect of TNF-alpha levels on thyroid hormone kinetics is the one by Ongphiphadhanakul et al. These investigators found that TNF-alpha might inhibit the peripheral generation of T_3_ from T_4_ and act as an autocrine factor by decreasing thyroidal generation and release of T_3_ [33]. Our data is not in accordance with this finding, since no correlation was found between TNF-alpha levels and thyroid hormones. In the present study, we demonstrated a significant IL-10 and TNF-alpha release after thyroid hormone induction, while the increase of IL-6 was insignificant. Thus, our results do not support any role for IL-6 in the pathogenesis of thyrotoxicosis.

IL-6 acts as a systemic hormone [35] that may mediate the well-documented inhibitory effect of IL-1 on thyroid cell functions [36]. In regard to IL-6, our findings are in agreement with the data presented by Senturk et al. [29] and Burggraaf et al. [32], since we could not present a statistically significant difference between thyrotoxic rats and control subjects. We suggest that this may be attributed to the duration of thyroid hormone induction or the consequence that since IL-6 can be bounded to plasma proteins, which may camouflage IL-6 immunoreactivity in serum, as previously suggested [29].

In contrast to IL-6, IL-10 is one of the most potent anti-inflammatory cytokines [37]. IL-10 interferes with cellular immunity in several ways and is known to attenuate the expression and/or production of proinflammatory cytokines [38]. TNF-alpha is expressed as one of these cytokines [39]. Our data did not support this, since both IL-10 and TNF-alpha levels were elevated after T_4_ induction. Interestingly, proinflammatory stimuli like TNF-alpha enhance its secretion without any influence of IL-6 [40]. There is a lack of evidence regarding IL-10 and nonautoimmune thyroid disorders. Data presented by de la Vega et al. supported that Il-10 levels in multinodular glands did not reach a significant difference versus normal subjects [41]. But the study population they studied was only 15, where each group consisted of 5 patients.

The link between free oxygen radicals and inflammatory processes in hyperthyroidism was studied previously by Fernández et al. [27]. They addressed that the effects of thyroid hormones on the expression of nuclear factor kappa B (NF-*κ*B) ligand are involved in the cytokine response seen in hyperthyroidism. There is evidence that T_3_ administration to rats show induced expression of NF-*κ*B-responsive genes for TNF-alpha and IL-10 [13]. This was correlated with increases in the serum levels of the cytokines in the same study.

Even though deleterious effects of NO^•^ in oxidative processes [42], its production is accepted to be induced by proinflammatory cytokines [43]. In our model, thyrotoxicosis resulted in obvious increased NO^•^x levels, and there was a correlation between NO^•^x and cytokine levels. This correlation suggests a link between NO and cytokine response in the thyrotoxic rat. This might simply mean that while a probable contributory role for these cytokines is suggested, it seems not to be the only factor involved in the complex pathogenesis in thyrotoxicity-related oxidative stress.

Melatonin, after discovered as a free radical scavenger in 1993 [44], has been reported to be protective in various models of oxidative stress, both through its free radical scavenging effect as well as by directly increasing antioxidant activity [45]. Although melatonin has been proposed to be useful for the prevention of oxidative stress during hyperthyroidism [23, 46], to our knowledge, there has been no report studying the effects of antioxidative therapy on serum concentrations of IL-6, IL-10, and TNF-alpha in the thyrotoxic rat. The hypothesis that melatonin is protective against free oxygen radicals was further supported by our observation that after melatonin treatment, increased MDA, GSH, and NO^•^x levels decreased significantly. Besides, thyroid hormone and TNF-alpha levels after melatonin treatment were lower than in untreated rats, suggesting an inhibitory effect of melatonin on T_3_, T_4_, and TNF-alpha. The decrement in TNF-alpha levels did not relate with the decrease of thyroid homone levels, suggesting a direct antioxidant effect of melatonin on TNF-alpha. Studies on the hypothesis of an inhibitory effect of melatonin on thyroid hormones documented contradictory results [47, 48]. In fact, melatonin is known with its stimulating effect on antioxidative enzymes, such as GR and GPx [49]. These enzymes convert oxidized glutathione to GSH, leading to increased GSH activity. However, in our study, melatonin administration did not result in increased GR and GPx levels. Moreover, it resulted in decreased GSH. This paradox might be attributed to differences in type and manner of melatonin administration. Melatonin suggests an immunotherapeutic potential, since it inhibits TNF-alpha. Whatever the mechanism is, the fact that melatonin counteracts the increase of TNF can help toward an understanding of the complex nature of TNF induction in hyperthyroidism.

Collectively, data presented in this work support a link between thyroid hormone-induced oxidative stress and cytokine response. It provides new insights into the role of IL-10 and TNF-alpha in thyrotoxicosis. Therapy for increased oxidative stress with melatonin significantly reduces triiodothyronine, thyroxine, IL-10, and TNF-alpha levels. The fact that circulating cytokines might differ from those in different tissues is a limitation of this study. Although the expression of cytokines seems to be involved with thyrotoxicosis-induced oxidative stress, this relation remains to be studied in future qualitative studies.

## Figures and Tables

**Table 1:** Comparison of thyroid function tests.

	*n*	Group A	Group B	Group C
FT_3_ (pg/mL)	7	1.49 ± 0.26	8.68 ± 3.69*	3.28 ± 2.98**
FT_4_ (ng/dL)	7	1.44 ± 0.44	4.62 ± 2.18*	1.94 ± 1.22**
TSH (*μ*IU/mL)	7	0.25 ± 0.02	0.15 ± 0.01	0.20 ± 0.36

Values were expressed as mean ± standard deviation. *P < .01; significant when compared with Group A. **P < .01; significant when compared with Group B.

**Table 2:** Comparison of MDA, GSH, GR, GPx, and NO^•^ derivate levels.

Group	*n*	MDA (*μ*mol/L)	GSH (mg/dL)	GR (U/g Hb)	GPx (U/g Hb)	NO (*μ*M/L)
A	7	3.42 ± 1.47	0.26 ± 0.06	0.83 ± 0.34	4.02 ± 1.51	6.72 ± 1.55
B	7	4.80 ± 1.23*	0.43 ± 0.06*	1.05 ± 0.39	3.48 ± 0.76	66.0 ± 58.54*
C	7	2.31 ± 0.71**	0.35 ± 0.07**	0.78 ± 0.31	3.88 ± 1.17	47.11 ± 15.18**

Values were expressed as mean ± standard deviation. *P < .01; significant when compared with Group A. **P < .01; significant when compared with Group B.

**Table 3:** Comparison of plasma cytokine levels.

Group	*n*	IL-6 (pg/mL)	IL-10 (pg/mL)	TNF-alpha (ng/mL)
A	7	324.66 ± 26.28	33.53 ± 10.03	0.76 ± 0.04
B	7	345.99 ± 109.20	51.99 ± 17.43*	0.88 ± 0.1*
C	7	333.33 ± 38.92	56.00 ± 10.33	0.75 ± 0.05**

Values were expressed as mean ± standard deviation. *P < .01; significant when compared with Group A. **P < .01; significant when compared with Group B.

## References

[1] Videla LA (2000). Energy metabolism, thyroid calorigenesis, and oxidative stress: functional and cytotoxic consequences. *Redox Report*.

[2] Fernández V, Videla LA (1993). 3, 3′,5-triiodothyronine-induced hepatic respiration: effects of desferrioxamine and allopurinol in the isolated perfused rat liver. *Toxicology Letters*.

[3] Bianchi G, Solaroli E, Zaccheroni V (1999). Oxidative stress and anti-oxidant metabolites in patients with hyperthyroidism: effect of treatment. *Hormone and Metabolic Research*.

[4] Magsino CH, Hamouda W, Ghanim H, Browne R, Aljada A, Dandona P (2000). Effect of triiodothyronine on reactive oxygen species generation by leukocytes, indices of oxidative damage, and antioxidant reserve. *Metabolism: Clinical and Experimental*.

[5] Mogulkoc R, Baltaci AK, Oztekin E, Sivrikaya A, Aydin L (2006). Effects of hyperthyroidism induced by L-thyroxin administration on lipid peroxidation in various rat tissues. *Acta Biologica Hungarica*.

[6] Karbownik M, Lewinski A (2003). The role of oxidative stress in physiological and pathological processes in the thyroid gland; possible involvement in pineal-thyroid interactions. *Neuroendocrinology Letters*.

[7] Ali MH, Schlidt SA, Chandel NS, Hynes KL, Schumacker PT, Gewertz BL (1999). Endothelial permeability and IL-6 production during hypoxia: role of ROS in signal transduction. *American Journal of Physiology*.

[8] Dröge W (2002). Free radicals in the physiological control of cell function. *Physiological Reviews*.

[9] Haddad JJ, Safieh-Garabedian B, Saadé NE, Land SC (2001). Thiol regulation of pro-inflammatory cytokines reveals a novel immunopharmacological potential of glutathione in the alveolar epithelium. *Journal of Pharmacology and Experimental Therapeutics*.

[10] Kosmidou I, Vassilakopoulos T, Xagorari A, Zakynthinos S, Papapetropoulos A, Roussos C (2002). Production of interleukin-6 by skeletal myotubes: role of reactive oxygen species. *American Journal of Respiratory Cell and Molecular Biology*.

[11] Schreck R, Rieber P, Baeuerle PA (1991). Reactive oxygen intermediates as apparently widely used messengers in the activation of the NF-*κ*B transcription factor and HIV-1. *The EMBO Journal*.

[12] Blackwell TS, Blackwell TR, Holden EP, Christman BW, Christman JW (1996). In vivo antioxidant treatment suppresses nuclear factor-*κ*B activation and neutrophilic lung inflammation. *Journal of Immunology*.

[13] Tapia G, Fernández V, Varela P, Cornejo P, Guerrero J, Videla LA (2003). Thyroid hormone-induced oxidative stress triggers nuclear factor-*κ*B activation and cytokine gene expression in rat liver. *Free Radical Biology and Medicine*.

[14] Ohkawa H, Ohishi N, Yagi K (1979). Assay for lipid peroxides in animal tissues by thiobarbituric acid reaction. *Analytical Biochemistry*.

[15] Tietze F (1969). Enzymic method for quantitative determination of nanogram amounts of total and oxidized glutathione: applications to mammalian blood and other tissues. *Analytical Biochemistry*.

[16] Racker E, Colowick SP, Kaplan NO (1955). Glutathione reductase (liver and yeast). *Methods in Enzymology*.

[17] Drabkin D, Austin H (1935). Spectropho-tometric studies: preparations from washed blood cells. *The Journal of Biological Chemistry*.

[18] Paglia DE, Valentine WN (1967). Studies on the quantitative and qualitative characterization of erythrocyte glutathione peroxidase. *The Journal of Laboratory and Clinical Medicine*.

[19] Navarro-Gonzálvez JA, García-Benayas C, Arenas J (1998). Semiautomated measurement of nitrate in biological fluids. *Clinical Chemistry*.

[20] Videla LA, Correa L, Rivera M, Sir T (1993). Zymosan-induced luminol-amplified chemoluminescence of whole blood phagocytes in experimental and human hyperthyroidism. *Free Radical Biology and Medicine*.

[21] Fernández V, Videla LA (1995). On the mechanism of thyroid hormone-induced respiratory burst activity in rat polymorphonuclear leukocytes. *Free Radical Biology and Medicine*.

[22] Mohamadin AM, Hammad LNA, El-Bab MF, Abdel Gawad HS (2007). Attenuation of oxidative stress in plasma and tissues of rats with experimentally induced hyperthyroidism by caffeic acid phenylethyl ester. *Basic & Clinical Pharmacology & Toxicology*.

[23] Baydas B, Meral I (2005). Effects of melatonin on lipid peroxidation and anti-oxidant enzyme activity in rats with experimentally induced hyperthyroidism. *Clinical and Experimental Pharmacology and Physiology*.

[24] Bartalena L, Tanda ML, Piantanida E, Lai A (2003). Oxidative stress and Graves' ophthalmopathy: in vitro studies and therapeutic implications. *BioFactors*.

[25] Borges F, Fernande E, Roleira F (2002). Progress towards the discovery of xanthine oxidase inhibitors. *Current Medicinal Chemistry*.

[26] Mogulkoc R, Baltaci AK, Oztekin E, Aydin L, Tuncer I (2005). Hyperthyroidism causes lipid peroxidation in kidney and testis tissues of rats: protective role of melatonin. *Neuroendocrinology Letters*.

[27] Fernández V, Videla LA, Tapia G, Israel Y (2002). Increases in tumor necrosis factor-*α* in response to thyroid hormone-induced liver oxidative stress in the rat. *Free Radical Research*.

[28] Mysliwiec J, Zbucki R, Winnicka MM (2007). A crucial role of interleukin-6 in the pathogenesis of thyroxicosis-related disturbances of bone turnover in mice. *Hormone and Metabolic Research*.

[29] Senturk T, Kozaci LD, Kok F, Kadikoylu G, Bolaman Z (2003). Proinflammatory cytokine levels in hyperthyroidism. *Clinical and Investigative Medicine*.

[30] Siddiqi A, Monson JP, Wood DF, Besser GM, Burrin JM (1999). Serum cytokines in thyrotoxicosis. *The Journal of Clinical Endocrinology & Metabolism*.

[31] Sekeroglu MR, Altun ZB, Algün E (2006). Serum cytokines and bone metabolism in patients with thyroid dysfunction. *Advances in Therapy*.

[32] Burggraaf J, Lalezari S, Emei JJ (2001). Endothelial function in patients with hyperthyroidism before and after treatment with propranolol and thiamazol. *Thyroid*.

[33] Ongphiphadhanakul B, Fang SL, Tang K-T, Patwardhan NA, Braverman LE (1994). Tumor necrosis factor-*α* decreases thyrotropin-induced 5′-deiodinase activity in FRTL-5 thyroid cells. *European Journal of Endocrinology*.

[34] Çelik I, Akalin S, Erbaş T (1995). Serum levels of interleukin 6 and tumor necrosis factor-*α* in hyperthyroid patients before and after propylthiouracil treatment. *European Journal of Endocrinology*.

[35] Boelen A, Platvoet-Ter Schiphorst MC, Wiersinga WM (1993). Association between serum interleukin-6 and serum 3,5,3′-triiodothyronine in nonthyroidal illness. *The Journal of Clinical Endocrinology & Metabolism*.

[36] Rasmussen AK, Feldt-Rasmussen U, Bendtzen K (1993). The effect of interleukin-1 on the thyroid gland. *Autoimmunity*.

[37] Bartalena L, Brogioni S, Grasso L, Velluzzi F, Martino E (1994). Relationship of the increased serum interleukin-6 concentration to changes of thyroid function in nonthyroidal illness. *Journal of Endocrinological Investigation*.

[38] Nicoletti F, Mancuso G, Ciliberti FA (1997). Endotoxin-induced lethality in neonatal mice is counteracted by interleukin-10 (IL-10) and exacerbated by anti-IL-10. *Clinical and Diagnostic Laboratory Immunology*.

[39] de Waal Malefyt R, Abrams J, Bennett B, Figdor CG, de Vries JE (1991). Interleukin 10(IL-10) inhibits cytokine synthesis by human monocytes: an autoregulatory role of IL-10 produced by monocytes. *Journal of Experimental Medicine*.

[40] Berger S, Siegert A, Denkert C, Köbel M, Hauptmann S (2001). Interleukin-10 in serous ovarian carcinoma cell lines. *Cancer Immunology, Immunotherapy*.

[41] de la Vega JR, Vilaplana JC, Biro A, Hammond L, Bottazzo GF, Mirakian R (1998). IL-10 expression in thyroid glands: protective or harmful role against thyroid autoimmunity?. *Clinical and Experimental Immunology*.

[42] Davis KL, Martin E, Turko IV, Murad F (2001). Novel effects of nitric oxide. *Annual Review of Pharmacology and Toxicology*.

[43] Nussler AK, Di Silvio M, Billiar TR (1992). Stimulation of the nitric oxide synthase pathway in human hepatocytes by cytokines and endotoxin. *Journal of Experimental Medicine*.

[44] Tan DX, Chen LD, Poeggeler B, Manchester LC, Reiter RJ (1993). Melatonin: a potent, endogenous hydroxyl radical scavenger. *Endocrine Journal*.

[45] Hardeland R (2005). Antioxidative protection by melatonin: multiplicity of mechanisms from radical detoxification to radical avoidance. *Endocrine*.

[46] Mogulkoc R, Baltaci AK, Oztekin E, Aydin L, Sivrikaya A (2006). Melatonin prevents oxidant damage in various tissues of rats with hyperthyroidism. *Life Sciences*.

[47] Baltaci AK, Mogulkoc R, Kul A, Bediz CS, Ugur A (2004). Opposite effects of zinc and melatonin on thyroid hormones in rats. *Toxicology*.

[48] Ashley PF, Frank LA, Schmeitzel LP, Bailey EM, Oliver JW (1999). Effect of oral melatonin administration on sex hormone, prolactin, and thyroid hormone concentrations in adult dogs. *Journal of the American Veterinary Medical Association*.

[49] Okatani Y, Wakatsuki A, Shinohara K, Kaneda C, Fukaya T (2001). Melatonin stimulates glutathione peroxidase activity in human chorion. *Journal of Pineal Research*.

